# Prehospital neuroprotective intervention: A critical imperative evidenced by the FRONTIER trial outcomes

**DOI:** 10.1515/jtim-2025-0037

**Published:** 2025-07-30

**Authors:** Min Zhao, Yuchuan Ding, Xunming Ji, Wenbo Zhao

**Affiliations:** Department of Neurology, Xuanwu Hospital, Capital Medical University, Beijing, China; Department of Neurosurgery, Wayne State University School of Medicine, Detroit, MI, USA; Beijing Institute for Brain Disorders, Capital Medical University, Beijing, China; Beijing Key Laboratory of Hypoxic Conditioning Translational Medicine, Xuanwu Hospital, Capital Medical University, Beijing, China; Stroke Research Group, Department of Clinical Neuroscience, University of Cambridge, Cambridge CB2 3PT, UK

Acute ischemic stroke (AIS), primarily caused by cerebral artery occlusion, is a time-sensitive emergency requiring prompt intervention to restore blood flow. Reperfusion therapy is the cornerstone of treatment, while neuroprotection holds promise as an adjunct to improve out co me s. ^[1, 2]^ Des pi te promising preclinical evidence, the clinical efficacy of specific neuroprotective agents remains uncertain, driving ongoing research efforts.^[3]^ Nerinetide, a selective post-synaptic density-95 inhibitor, exhibits neuroprotective effects in preclinical trials by reducing glutamate excitotoxicity and limiting nitric oxide overproduction during ischemia.^[4]^ Recent insights into neuroprotection have emerged from the nerinetide trial series, notably the phase 3 ESCAPE-NEXT trial and the FRONTIER trial.^[5,6]^ The ESCAPE-NEXT trial found no significant benefit of nerinetide as an adjunct to endovascular thrombectomy without intravenous thrombolysis in AIS patients. In contrast, the FRONTIER trial suggested that prehospital nerinetide administration may improve functional outcomes in certain AIS subgroups treated with reperfusion therapy, despite neutral primary endpoint results. The superior results of nerinetide observed in the FRONTIER trial may stem from its ultra-early intervention window, with nerinetide administered at a median of 64 minutes of symptom onset, which was further supported by the post-hoc individual patient-level meta-analysis pooled data from the ESCAPE-NA1, ESCAPE-NEXT, and FRONTIER trials, focusing on patients treated within 3 hours of stroke onset and selected for reperfusion without previous thrombolysis, and showing that nerinetide was associated with improved functional outcomes.^[7]^ Ultra-early neuroprotective intervention, as demonstrated in preclinical studies, maximizes ischemic penumbral preservation and may extend the therapeutic window for reperfusion.

The ESCAPE-NEXT and FRONTIER trials suggest that early neuroprotective treatment before reperfusion therapy may be effective. This approach is based on the gradual expansion of the infarct core, with early treatment potentially limiting core growth and optimizing conditions for reperfusion, thereby improving outcomes.^[8]^ However, this strategy raises a key question: how does the neuroprotective agent reach the ischemic tissue before the occluded vessel is recanalized? After vessel occlusion, the blood supply of infarcted region relies on collateral circulation, which significantly affects infarct volume and ischemic penumbra size before recanalization. The efficacy of neuroprotective agents is also influenced by collateral circulation, as drug delivery depends on collateral blood flow, introducing a potential confounding factor.^[9]^ Patients with competent collateral circulation benefit from dual advantage, enhanced drug delivery to penumbral tissue, and robust collateral circulation naturally tend to experience better prognoses. Conversely, collateral failure creates a “double deficit”—compromised therapeutic agent penetration combined with accelerated penumbral reduction. Consequently, in the FRONTIER trial, supplementary analysis of the data by stratifying it according to the quality of collateral circulation could offer deeper, more nuanced insights into the true effectiveness of early neuroprotective treatment. In resource-limited settings where advanced imaging is unavailable, primary collateral status assessment could be based on clinical predictors, such as age, sex, and vascular comorbidities (*e.g*., hypertension, diabetes mellitus, *etc*.).

The FRONTIRE trial yielded positive results, suggesting that interventions aimed at protecting the ischemic penumbra represent a highly promising therapeutic avenue for patients with AIS who are candidates for reperfusion therapy.^[10]^ Several neuroprotective strategies, including normobaric oxygen (NBO) and remote ischemic conditioning, have been explored in prehospital settings, with multiple clinical trials currently in progress. Preclinical studies indicate that neuroprotective approaches can stabilize the ischemic penumbra before vessel recanalization, but confirming this in clinical practice remains challenging. In prehospital settings, distinguishing between ischemic, hemorrhagic, or mimicking strokes is challenging. Even with a mobile stroke unit to exclude hemorrhagic stroke, confirming large vessel occlusion and ischemic penumbra remains difficult. Considering the current pre-hospital emergency care system, studying neuroprotective effects on the ischemic penumbra in patients diagnosed with large vessel occlusion and ischemic penumbra via computed tomography angiography (CTA) and computed tomography perfusion (CTP) at a primary stroke center, followed by transfer for thrombectomy, may be more feasible. Studies on interhospital transfer utilizing NBO are currently being designed with this approach in mind.

**Figure 1:**
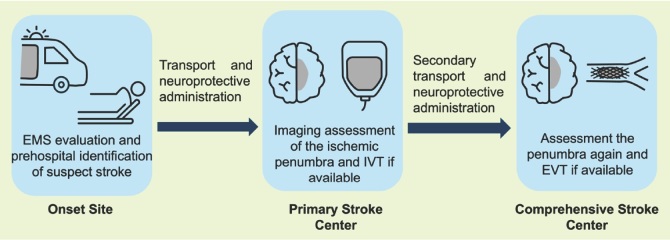
Neuroprotective agents are administered during patient transport. Quantitative comparisons of ischemic penumbravolume before and after transport can then reveal the stabilizing effect of neuroprotective agents on the penumbra. EMS indicates emergency medical services; IVT: intravenous thrombolysis; EVT: endovascular treatment.

The ESCAPE-NEXT and FRONTIER trials, supported by the meta-analysis, underscore the potential of nerinetide as a neuroprotective agent, particularly when administered ultra-early before vascular recanalisation. However, the limitations of the studies still warrant attention. Interpretative power is limited by the small sample size of ischemic stroke patients, mainly due to prehospital diagnostic limitations. Future studies with larger cohorts and blood-based biomarkers may be needed to validate these findings. Future research should focus on optimizing the timing and delivery of nerinetide, potentially exploring prehospital administration protocols and integrating them with mobile stroke units. Stratifying analyses by collateral circulation status, as suggested in related discussions, could yield deeper insights into patient subgroups most likely to benefit. Additionally, investigating interactions with thrombolytic agents and refining inclusion criteria based on imaging (*e.g*., CTA, CTP) could enhance trial outcomes (Figure 1).
